# *In vitro* and *in vivo* characterization of a West Nile virus MAD78 infectious clone

**DOI:** 10.1007/s00705-014-2176-2

**Published:** 2014-07-15

**Authors:** Katherine L. Hussmann, Rianna Vandergaast, Susan Park Ochsner, Albert C. Huang, Michael Gale, Brenda L. Fredericksen

**Affiliations:** 1 Maryland Pathogen Research Institute, University of Maryland-College Park, College Park, USA; 2 Department of Cell Biology and Molecular Genetics, University of Maryland-College Park, College Park, USA; 3 Department of Immunology, University of Washington School of Medicine, Seattle, WA USA; 4 Present Address: Department of Veterinary Medicine, University of Maryland-College Park, College Park, USA; 5 Present Address: Aids Review Branch, National Institute of Allergy and Infectious Diseases, National Institutes of Health, DHHS, Bethesda, MD 20817 USA

**Keywords:** West Nile virus, WNV, Infectious clone, WNV-MAD78, Pathogenicity

## Abstract

The viral determinants governing the varied neuropathogenicity of different West Nile virus (WNV) strains are poorly understood. Here, we generated an infectious clone (WNV-MAD^IC^) of the non-pathogenic strain WNV-MAD78 and compared its replication to that of parental WNV-MAD78 and a WNV-MAD78 infectious clone (WNV-MAD^TX-UTRs^) containing the 5′ and 3′ untranslated regions (UTRs) of the pathogenic strain WNV-TX. All three viruses replicated at similar rates and caused similar lethality in mice. Thus, the infectious clone is indistinguishable from parental virus in replication and neurovirulence, and the UTRs alone do not account for the increased virulence of WNV-TX compared to WNV-MAD78.

West Nile virus (WNV) is a member of the genus *Flavivirus*, family *Flaviviridae*. In Africa, Asia, and the Middle East, WNV is endemic, and infection is primarily asymptomatic or associated with a mild febrile illness known as WN fever. However, recent outbreaks of WNV in the western hemisphere have occurred with a significantly higher incidence of severe neurological disease [1–5]. The viral determinants responsible for the increased virulence of these emerging WNV strains are poorly understood.

The naturally occurring diversity in the neuropathogenicity among strains of WNV provides an excellent model system to define virulence determinants of emergent strains. To facilitate comparative pathogenicity studies, we generated a complete full-length infectious clone of the lineage 2 African isolate WNV-MAD78. Since WNV-MAD78 is non-pathogenic in mice when inoculated in the periphery [6, 7], it is a useful strain for comparison studies with pathogenic lineage 1 strains such as WNV-NY99 or WNV-TX02, for which infectious clones are also available.

Although the sequence of the first 10,866 nucleotides, representing nearly the entire genome, of WNV-MAD78 was reported previously (accession DQ176636), the precise length and sequence of the 3′ end of the genome was not determined. Therefore, we sequenced the 3′ end of WNV-MAD78 RNA extracted from infected A549 cells. TRIzol (Invitrogen)-extracted RNA was treated with Terminator Exonuclease (Epicentre) to selectively remove rRNA. Since the WNV genome lacks a polyA tail, the extracted RNA was polyadenylated with polyA polymerase (NEB) and then used as a template for reverse transcription (RT) with an oligo-dT primer. The 3′ end of the resulting cDNA was PCR-amplified using the oligo-dT primer and a sense primer corresponding to position 10743 within the WNV-MAD78 genome. Sequence analysis identified 20 nucleotides immediately adjacent to the added polyA nucleotides that aligned with the 3′ end of another lineage 2 strain of WNV, WNV-956, suggesting that this sequence represented the exact 3′ end of WNV-MAD78. To confirm the sequence of the WNV-MAD78 3′UTR, we repeated the amplification of this region using the 10743 primer and an antisense primer complimentary to the exact 3′ end of the WNV-MAD78 genome. Sequence analysis of the resulting RT-PCR product identified a total of 97 nucleotides that were not reported previously (Fig. 1a and accession number KJ909513). Alignment of the complete 3′ UTR sequence of WNV-MAD78 with that of other WNV strains indicated that the WNV-MAD78 3′ UTR diverges from most lineage 1 and 2 strains (Fig. 1b). In contrast, the WNV-MAD78 5′UTR is highly conserved (Fig. 1b).

**Fig. 1:**
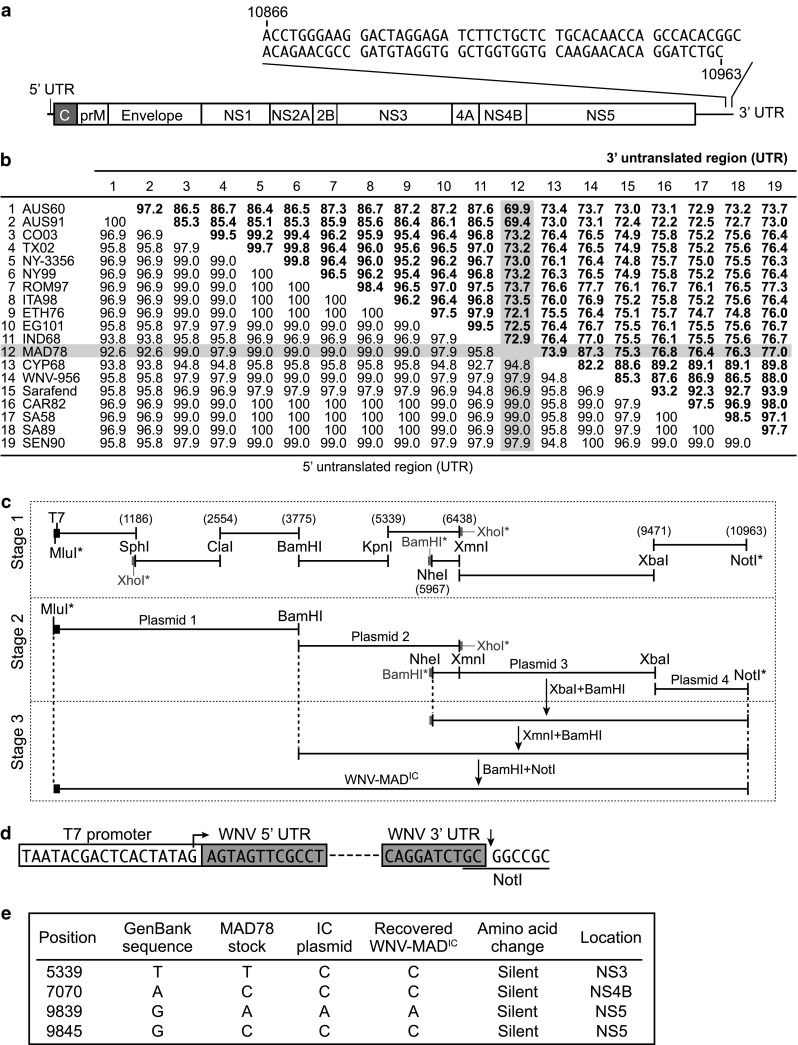
Generation of a WNV-MAD78 infectious clone. (**a**) Scale representation of WNV-MAD78. The last 97 nucleotides of the genome, representing the additional portion sequenced here, are indicated. (**b**) Percent identity of the 5′ and 3′ UTRs among WNV strains. Sequences were downloaded from GenBank, and ClustalW Alignment was performed with MacVector version 10.0.2 using the entire UTR sequences. Values represent percent identities among strains for the 3′ UTR (bold text) or 5′ UTR (regular text). The strains used (with accession numbers indicated in parentheses) are as follows: AUS60 (GQ851602), AUS91 (GQ851603), CO03 (DQ164203), TX02 (DQ164198), NY-3356 (AF404756), NY99 (AY842931), ROM97 (AF260969), ITA98 (AF404757), ETH76 (AY603654), EG101 (EU081844), IND68 (EU249803), MAD78 (DQ176636), CYP68 (GQ903680), WNV-956 (NC_001563), Sarafend (AY688948), CAR82 (DQ318020), SA58 (EF429200), SA89 (EF429197), and SEN90 (DQ318019). Strains 1 to 11 are lineage 1 and strains 12 to 19 are lineage 2. (**c**) Construction of the infectious clone. RT-PCR was carried out on parental WNV-MAD78 RNA to generate the eight cDNAs shown in Stage 1. Nucleotide boundaries are numbered based on parental WNV-MAD78. Restriction sites inserted during RT-PCR, which are not present in the genome, are indicated (*), and those shown in gray were not present in the final full-length construct. In Stage 2, the indicated fragments were subcloned into the four plasmids indicated (Plasmid 1, pWSK29 [9]; Plasmid 2, pEGFP (Clonetech); Plasmid 3, pBKSM (Stratagene); Plasmid 4, pWSK129 [9]). In Stage 3, the WNV-MAD78 regions of Plasmids 1–4 were combined in the order indicated to generate the full-length WNV-MAD^IC^. Arrows indicate restriction digests. (**d**) Annotated sequence of WNV-MAD^IC^ ends. On the left, the sequence and location of the T7 promoter (white box) relative to the WNV 5′UTR (grey box) is indicated. The arrow indicates the site of transcription initiation. On the right, the placement of the *NotI* site (underlined nucleotides) at the end of the WNV 3′ UTR (grey box) is shown, with an arrow representing the position of endonuclease cleavage. (**e**) Table of sequence differences between WNV-MAD^IC^ and parental WNV-MAD78. Differences between our WNV-MAD78 stock, the recovered WNV-MAD^IC^ virus, the WNV-MAD^IC^ plasmid, and the published WNV-MAD78 GenBank sequence (accession DQ176636) are indicated

To generate a full-length infectious clone of WNV-MAD78, we utilized the strategy outlined in Fig. 1c. First, total RNA extracted from WNV-MAD78-infected Vero cells was used as template for RT-PCR to generate eight WNV-MAD78 cDNAs spanning the entire virus genome (Fig. 1c, Stage 1). During PCR, an *MluI* restriction site followed by the T7 promoter was inserted immediately upstream of the 5′ end of the genome (Fig. 1d). Several other restriction sites were engineered at various locations within the cDNAs to facilitate cloning but were not incorporated into the final construct. Additionally, a *NotI* restriction site was inserted at the 3′ end of the genome (Fig. 1d). Each cDNA was blunt-end ligated into the cloning vector pVL-blunt (a kind gift from Vincent Lee) [8]. In Stage 2, the eight WNV fragments were subcloned as indicated in Fig. 1c to generate four plasmids encoding the entire genome. The full-length infectious clone (pWNV-MAD^IC^; accession number KJ909514) was generated by assembling the four WNV segments into the very-low-copy plasmid pWSK29 [9] as outlined in Fig. 1c, Stage 3. Sequencing of the final full-length construct identified four silent mutations compared to the previously published WNV-MAD78 sequence (Fig. 1e). To determine if these mutations were introduced during the cloning process, we sequenced the corresponding regions of our WNV-MAD78 stock. Of the four mutations noted, three were present in our WNV-MAD78 stock (Fig. 1e), indicating that only one (T5339C) arose during the cloning process.

To generate virus, linear pWNV-MAD^IC^ was used as template for *in vitro* transcription, and 12 μg of the resulting RNA was used to transfect 1 × 10^6^ Vero cells using the Neon® Transfection System (Invitrogen) set to the following conditions: 1150 V, 20 ms, and 2 pulses. After seven days, culture supernatant containing WNV-MAD^IC^ was collected and subsequently passaged once in Vero cells to generate a working stock. The presence of a cytosine nucleotide at position 5339 within the recovered virus (Fig. 1e) confirmed that it was WNV-MAD^IC^ and not parental WNV-MAD78.

To assess the biological properties of WNV-MAD^IC^, we compared the growth kinetics of the recovered virus to that of the parental strain. Vero cells were infected with WNV-MAD78 or WNV-MAD^IC^ and infectious particle production assessed by plaque assay (Fig. 2a). Similar levels of infectious particles were observed at all times, with peak levels occurring at 48 h after infection for both viruses (Fig. 2a). Moreover, plaques for both viruses developed at similar rates on Vero cells and were visible at 6 days post-inoculation. To assess virulence, wild-type C57BL/6 and interferon receptor knockout (Ifnar-/-) mice, which are resistant and highly susceptible, respectively, to WNV-MAD78 [7], were inoculated subcutaneously with 100 PFU of WNV-MAD78 or WNV-MAD^IC^ (Fig. 2b). No differences in survival were observed. In wild-type mice, neither strain caused mortality or weight loss, though one mouse inoculated with WNV-MAD^IC^ showed mild signs of disease. In contrast, in the absence of IFN signaling, infection with either strain was 100 % lethal. Thus, the virus recovered from the infectious clone was indistinguishable from the parental strain.

**Fig. 2:**
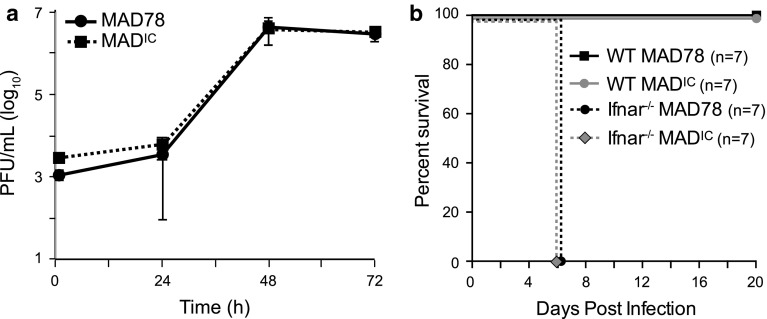
WNV-MAD^IC^ displays similar biological properties to parental WNV-MAD78. (**a**) Vero cells were inoculated with WNV-MAD78 or WNV-MAD^IC^ (MOI = 0.05). Culture supernatants were collected at the indicated times and the concentration of virus was determined by plaque assay on Vero cells. Values represent the average number of plaque-forming units (PFU) per mL of supernatant (+/- standard deviation) from three independent experiments. (**b**) Eight- to twelve-week-old C57BL/6 or Ifnar -/- mice were infected with WNV-MAD78 or WNV-MAD^IC^ (n = 7) by subcutaneous injection of 100 PFU into the left rear footpad. Mice were monitored daily and euthanized when body weight loss was >20 % or they reached clinical scores of 4 or above (1, no paresis; 2, mild paresis; 3, frank paresis; 4, severe paresis; 5, true paresis; 6, moribund)

Recently, Suthar *et al.* generated a similar infectious clone, herein referred to as WNV-MAD^TX-UTRs^, which contains the 5′ and 3′ UTRs of a virulent WNV strain, WNV-TX [10]. The WNV-TX UTRs have notable differences from those of WNV-MAD78. Within the 5′ UTR, WNV-TX contains an extra adenosine at position 51, and the WNV-TX 3′UTR is 78 nucleotides longer and shares only 73.2 % sequence identity with that of WNV-MAD78 (Fig. 1b). Specific sequences and structural folds within the UTRs are critical for WNV replication [11–15], and previous work suggests that the UTRs are virulence determinants [16]. Thus, sequence differences among the UTRs of various WNV strains may alter the structure and function of these regions and thereby influence the pathogenicity of the resulting virus. To assess the effect of the TX-UTRs on WNV-MAD78 fitness, we compared WNV-MAD78, WNV-MAD^IC^, and WNV-MAD^TX-UTRs^ replication in A549 cells, human brain cortical astrocytes (HBCAs), and C6/36 cells, models for WNV infection within the periphery, the neurovascular unit, and the mosquito vector, respectively. In all cell types, similar titers were observed for all three viruses throughout the infection (Fig. 3a-c), consistent with the initial characterization of WNV-MAD^TX-UTRs^ within A549 cells [10]. Thus, the TX-UTRs are neither beneficial nor detrimental to WNV-MAD78 replication *in vitro*. However, we consistently observed that WNV-MAD^TX-UTRs^ plaques on Vero cells were larger than those of WNV-MAD^IC^ or parental WNV-MAD78 (Fig. 3d), suggesting that the presence of TX-UTRs may marginally enhance viral spread in some mammalian cells.

**Fig. 3:**
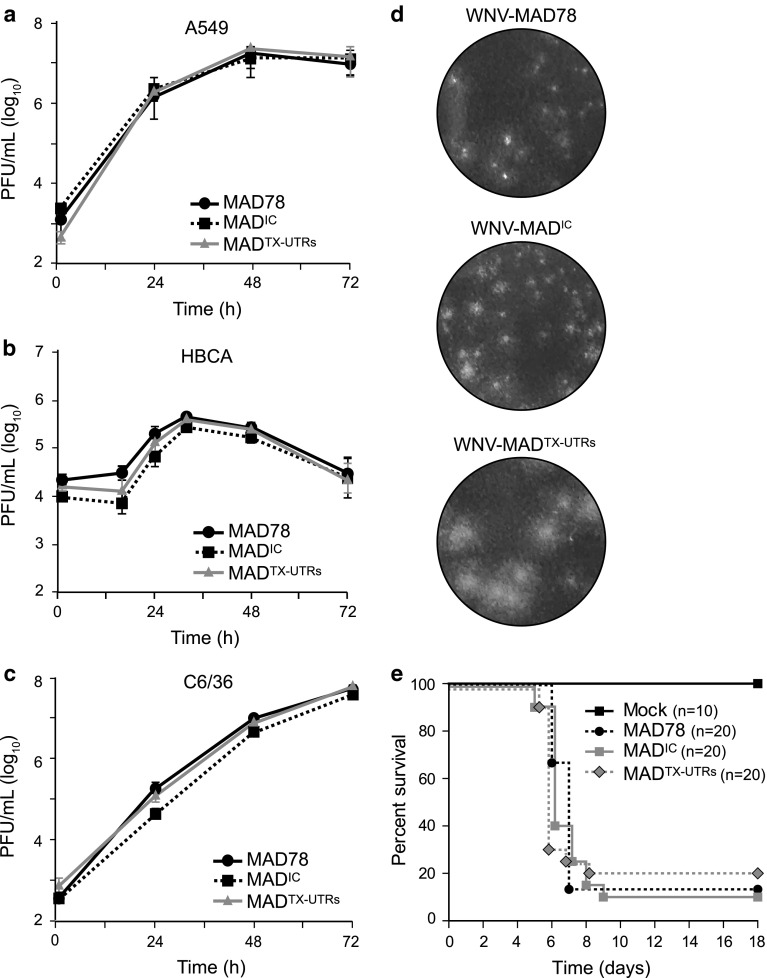
WNV-MAD78, WNV-MAD^IC^, and WNV-MAD^TX-UTRs^ exhibit similar replication and neurovirulence. (**a**-**c**) A549 (**a**) (MOI = 0.05), HBCA (**b**) (MOI = 0.01), or C6/36 (**c**) (MOI = 0.02) monolayers were inoculated with WNV-MAD78, WNV-MAD^IC^, or WNV-MAD^TX-UTRs^. Culture supernatants were collected and titers determined as in Fig. 2(a). (**d**) Vero monolayers were infected with ~50 pfu of WNV-MAD78, WNV-MAD^IC^, or WNV-MAD^TX-UTRs^. After 1 h, inoculums were removed and a 0.9 % agarose-media overlay was added. After 7 days, the monolayers were fixed with 4 % formaldehyde and stained with crystal violet. (**e**) Four-week-old Swiss Webster mice were inoculated intracranially with 10 pfu of WNV-MAD78 (n = 20), WNV-MAD^IC^ (n = 20), or WNV-MAD^TX-UTRs^ (n = 20) or mock inoculated (n = 10) with 20 μL of PBS. Mice were monitored daily and euthanized when body weight loss was >20 % or clinical scores indicated severe disease

When inoculated in the periphery, parental WNV-MAD78 and WNV-MAD^TX-UTRs^ are completely avirulent in mice [10]. In contrast, approximately 60 % of mice inoculated intracranially with parental WNV-MAD78 succumb to infection [17], making intracranial (IC) inoculation a good model for assessing virulence. To determine if the WNV-TX UTRs affected WNV-MAD78 virulence, we compared WNV-MAD78, WNV-MAD^IC^, and WNV-MAD^TX-UTRs^ in 4–week-old outbred Swiss Webster mice (Harlan Laboratories) inoculated intracranially with 10 pfu of virus or a sham PBS control. All viruses exhibited similar lethality (Fig. 3e, p = 0.766), with 80–90 % of infected animals succumbing to infection by 9 days after inoculation. Morbidity correlated directly with mortality, and no animals that lost weight or showed signs of disease recovered (data not shown). Thus, the virulence phenotypes of the infectious clones are indistinguishable from each other and from that of parental WNV-MAD78.

In summary, we have generated an infectious WNV-MAD78 clone (WNV-MAD^IC^) that contains the authentic UTRs of the viral RNA and is indistinguishable from parental WNV-MAD78 in replication and neurovirulence. Replacing the WNV-MAD78 UTRs with those of a pathogenic strain of WNV had no effect on the biological phenotype of WNV-MAD78. This finding is consistent with a study of another low-pathogenicity WNV strain, Kunjin virus, in which replacing both the Kunjin UTRs with those of WNV-NY did not enhance virulence in mice [16], though replacing the 5′ UTR alone did increase pathogenicity. Thus, divergence among the UTRs of various WNV strains may not be a determining factor in virulence, as long as they are maintained as a cognate pair.
